# Preliminary evidence for changes in frontoparietal network connectivity in the early abstinence period in alcohol use disorder: a longitudinal resting-state functional magnetic resonance imaging study

**DOI:** 10.3389/fpsyt.2023.1185770

**Published:** 2023-07-28

**Authors:** Jasper van Oort, Nancy Diazgranados, David T. George, Yvonne Horneffer, Melanie Schwandt, David Goldman, Reza Momenan

**Affiliations:** ^1^Clinical NeuroImaging Research Core, National Institute on Alcohol Abuse and Alcoholism, National Institute of Health, Bethesda, MD, United States; ^2^Office of the Clinical Director, National Institute on Alcohol Abuse and Alcoholism, National Institute of Health, Bethesda, MD, United States

**Keywords:** fMRI, addiction, alcohol use disorder, resting-state connectivity, recovery

## Abstract

The early abstinence period is a crucial phase in alcohol use disorder (AUD) in which patients have to find a new equilibrium and may start recovery, or conversely, relapse. However, the changes in brain functions during this key period are still largely unknown. We set out to study longitudinal changes in large-scale brain networks during the early abstinence period using resting-state scans. We scanned AUD patients twice in a well-controlled inpatient setting, with the first scan taking place shortly after admission and the second scan 4 weeks (±9 days) later near the end of the treatment period. We studied 37 AUD patients (22 males) and 27 healthy controls (16 males). We focused on three networks that are affected in AUD and underly core symptom dimensions in this disorder: the frontoparietal networks (left and right FPN) and default mode network (DMN). Both the whole brain and within network connectivity of these networks were studied using dual regression. Finally, we explored correlations between these brain networks and various neuropsychological and behavioral measures. In contrast to the controls (*Z* = −1.081, *p* = 0.280), the AUD patients showed a decrease in within left FPN connectivity (*Z* = −2.029, *p* = 0.042). However, these results did not survive a strict Bonferroni correction. The decrease in left FPN connectivity during the early abstinence period in AUD may reflect an initially upregulated FPN, which recovers to a lower resting-state connectivity level during subsequent weeks of abstinence. The AUD patients showed a trend for a positive association between the change in left FPN connectivity and trait anxiety (r_s_ = 0.303, *p* = 0.068), and a trend for a negative association between the change in left FPN connectivity and delay discounting (r_s_ = −0.283, *p* = 0.089) (uncorrected for multiple comparisons). This suggests that the FPN might be involved in top-down control of impulsivity and anxiety, which are important risk factors for relapse. Although there were no statistically significant results (after multiple comparison correction), our preliminary findings encourage further research into the dynamic neuroadaptations during the clinically crucial early abstinence period and could inform future study designs.

## 1. Introduction

Alcohol use disorder (AUD) is characterized by dysregulation across various neurobiological and neuropsychological domains (1–4). Although empirical studies suggest that both neurobiological and behavioral measures improve substantially after stopping alcohol use, the trajectories over which recovery takes place are still poorly understood (1). While drinking leads to adaptations to alcohol in AUD, a new equilibrium has to be found after stopping drinking (2, 5, 6). The initial weeks of abstinence mark a crucial period in which dynamic adaptations may help to start and subsequently sustain recovery, while failure to adapt may presage relapse (1). The importance of the early abstinence period is highlighted by studies showing that AUD patients are most likely to relapse in the first weeks following attempted abstinence (7, 8). Despite the importance of this early abstinence period, it is still largely unknown how brain function changes during this phase.

Functional brain networks can be identified *via* detection of patterns of synchronized activity between distributed brain regions (9), and have emerged as fundamental, dynamically organized elements of human brain function (10, 11). The frontoparietal networks (left and right FPN) are among the most investigated networks in addiction and are involved in higher-order cognitive processes and top-down cognitive control functions, including inhibition, emotion regulation, working memory, and cognitive flexibility (12–15). In addition, there is mounting evidence for the critical role of the default mode network (DMN) in addiction disorders (4). The DMN facilitates spontaneous and self-referential thought (16). Aberrant patterns in functional connectivity of the DMN have been observed across AUD and other substance use disorders and have been associated with impaired self-awareness, negative emotions, rumination, and craving (4).

Resting-state studies have shown that AUD is characterized by aberrant connectivity patterns of the FPN (17–19) and DMN (4). There is extensive evidence for differences in resting-state connectivity of the FPN between AUD patients and healthy individuals (14, 18, 20, 21). Several studies have shown increased resting-state connectivity of the FPN in abstinent AUD patients compared to healthy individuals, which is often interpreted as compensatory upregulation of the FPN for top-down control (14, 18, 20). In addition, various studies have found differences in DMN connectivity between abstinent AUD patients and healthy individuals (4, 17, 21–23), with among others reduced connectivity between the anterior and posterior DMN in AUD (21). Interestingly, a recent longitudinal resting-state study showed changes in network organization (including core regions of the DMN and FPN) in the period from one month abstinence to three month later, which were related to patterns in abstinence and relapse during this three month follow-up period (24). Although these various studies have significantly contributed to a better understanding of the neural mechanisms of AUD, relatively few studies focus on the first weeks of abstinence. Additionally, the overwhelming majority of the studies are cross-sectional, highlighting the need for more longitudinal studies.

In the studies that have been performed in the early abstinence period, differences have been found between AUD patients and healthy controls for both FPN and DMN connectivity (23, 25). In their cross-sectional study, Zhu and colleagues (23) showed increased connectivity within the FPN in AUD patients in the early abstinence period compared to the controls. When Zhu and colleagues analyzed the anterior DMN and posterior DMN separately they found increased within network connectivity in both these DMN subsystems (23). While there are no studies investigating changes in functional connectivity longitudinally during early abstinence, there are various longitudinal studies investigating changes in brain structure [e.g. (26–29)]. These studies have shown substantial recovery during the early abstinence period (26), with changes in both grey matter (26, 29) and white matter tracts during this phase (27, 28). Longitudinal studies of changes in functional network connectivity in the early abstinence phase can help to better characterize this dynamic and clinically critical period in which patients must cope with the challenges of finding a new equilibrium and staying abstinent (7, 8).

In this study, we set out to investigate the early abstinence period in moderately to severely ill AUD inpatients, from a large-scale functional network perspective, with a focus on the DMN and FPN. A better understanding of the mechanisms underlying the early recovery from AUD may ultimately help to identify biomarkers that may serve as targets for personalized treatments in the future, with the FPN for example being an important target for non-invasive neurostimulation treatments, like repetitive transcranial magnetic stimulation (rTMS) (4, 30, 31). Our main aim was to investigate changes in the connectivity of our networks of interest in AUD patients during this early abstinence period. Within this setup, we primarily focused on the changes within the networks of interest and expanded this to changes in connectivity patterns of these networks across the entire brain. We hypothesized that the AUD group would show an increase in within FPN connectivity during the early abstinence period (to support top-down control) (14, 18, 20), and an increase in within DMN connectivity, reflecting recovery toward more integrative functioning of the DMN (between, among others, the anterior and posterior DMN) (4, 21). Finally, we performed exploratory correlations to investigate how changes in connectivity are associated with various neuropsychological and behavioral measures related to alcohol use, executive functioning, impulsivity, and anxiety.

## 2. Methods and materials

### 2.1. Participants

The present study was conducted at the National Institute on Alcohol Abuse and Alcoholism (NIAAA). Participants in the Alcohol Use Disorder (AUD) group were adult individuals partaking in NIAAA’s inpatient treatment program at the National Institute of Health (NIH) Clinical Center (Bethesda, Maryland) between 2016 and 2018. Patients underwent alcohol detoxification if necessary. All AUD patients and controls were studied within the framework of a Natural History Protocol such that common data elements such as Structured Clinical Interviews for DSM (SCIDs) were collected. Healthy control participants were enrolled through the NIAAA Outpatient Clinic at the NIH Clinical Center in the same time frame. Unlike the AUD inpatients, control participants were not admitted to the inpatient ward during the study period. The human research protocols were approved by the NIH Institutional Review Board and all participants signed informed consent before participation.

Demographic and clinical characteristics [i.e., age, sex, years of education, race, and smoking status (32)] were collected. All participants were diagnosed according to the Diagnostic and Statistical Manual of Mental Disorders (DSM) (33). Since the present study took place during the transition period from DSM-IV to DSM-5 psychiatric diagnoses were made *via* either the Structured Clinical Interview for DSM-IV (SCID-IV-CT) (34) or the Structured Clinical Interview for DSM-5 Disorders (SCID-5-RV) (35). All AUD participants met criteria for either alcohol dependence (DSM-IV) or moderate-to-severe AUD (DSM-5) (36). A control group was included that was matched with the AUD group on age and sex.

All participants were between 30 and 60 years old and physically healthy. Patients were only included if they no longer experienced active withdrawal symptoms as determined by the Clinical Institute Withdrawal Assessment score (CIWA ≤8) (37). Exclusion criteria were: a history of neurological disorders of the central nervous system, cranial surgery, diabetes, history of significant head trauma, clinical or laboratory evidence of severe hepatic disease (i.e., ALT or AST > 5 times the upper normal level, INR > 2.0, total bilirubin >2.5 mg/dL, albumin <3.0 g/dL), a positive HIV test, current pregnancy (for women), contraindications related to the MRI scan (e.g., related to nonremovable ferrous metal in the body and claustrophobia), or positive breath alcohol test or urine drug test on the day of the MRI scan (except for benzodiazepines in the patients, as these are used to treat alcohol withdrawal during detoxification and might still show up in the urinary drug screen). Except for two patients, who still had positive urine tests for benzodiazepines at timepoint 1, all other urine tests were negative. The following additional exclusion criteria were used for the control group: a diagnosis of alcohol abuse or dependence (DSM-IV), AUD (DSM-5) or any other current DSM-IV or DSM 5 diagnosis *via* the SCID, use of any psychotropic medication on the day of scanning, more than seven standard drinks/week for females or fourteen standard drinks/week for males, and five or more binge drinking episodes (i.e., for males ≥5 standard drinks and for females ≥4 standard drinks on one occasion within 2 h) in the past 30 days (NB: while this allowed for binge drinking episodes in the controls, none of the included controls had any binge drinking episodes during this period).

### 2.2. Procedure

All participants were scanned twice to measure functional connectivity. The AUD patients were first scanned at treatment entry, within the first seven days of their admission to the treatment program and subsequent to acute withdrawal (Timepoint 1). The second scan (Timepoint 2) took place at the end of the inpatient treatment program (4 weeks ±9 days later). The control participants were also scanned twice, with a same interval between the two scans as for the patients. Abstinence in the AUD participants was ensured during the stay on the inpatient unit by regular monitoring including breath alcohol tests at least three times a day in the context of their treatment. The NIAAA’s treatment program for the AUD patients included group and individual therapy and pharmacological interventions when appropriate.

The complete magnetic resonance imaging (MRI) session consisted of multiple scans, of which we used the T1 structural and resting-state scan for the present study. The T1 structural scan was the first scan in the protocol and the resting-state scan the second scan. During acquisition of the resting-state data participants were instructed to lie still with their eyes open. Participants were instructed not to think about anything in particular. An MRI compatible eye-tracking device using infrared light was used to monitor whether the participants stayed awake during the resting-state scan.

### 2.3. fMRI data acquisition

All images were collected using a 3 Tesla MRI scanner (Siemens Magnetom Prisma) with a 32-channel head coil. T2*-weighted EPI BOLD-fMRI images were acquired for the resting-state scans, using an interleaved slice acquisition sequence (number of volumes: 315, number of slices = 36, TR = 2,000 ms, TE = 30 ms, flip angle = 90^0^, voxel size = 3.8 mm isotropic, slice gap = 0 mm, FOV = 240 mm, GRAPPA acceleration factor 2). High-resolution structural images (1.0 mm isotropic) were acquired using a T1-weighted MP-RAGE sequence (TE/TR = 1.63/2460 ms, flip angle = 5^0^, FOV = 288 × 288 × 208 mm, GRAPPA acceleration factor 2).

### 2.4. fMRI preprocessing

Preprocessing and statistical analyses were performed using FSL 6.0.5.2 (FMRIB, Oxford, UK). The resting-state scans were preprocessed using the FMRI Expert Analysis Tool (FEAT), which is part of the FMRIB Software Library (FSL) (38). To allow for T2* equilibration effects, the first five images of each resting-state scan were discarded. Furthermore, the preprocessing steps included brain extraction, motion correction, bias field correction, high-pass temporal filtering with a cut-off of 100 s, spatial smoothing with a 4 mm full width at half maximum (FWHM) Gaussian kernel, registration of functional images to high-resolution T1 using boundary-based registration and nonlinear registration to standard space (MNI152). We used ICA-based Advanced Removal of Motion Artifacts (ICA-AROMA) for further single-subject denoising (39). Participants were excluded from analyses if motion resulted in more than 3.8 mm (1 voxel) sudden relative mean displacement or translation.

### 2.5. fMRI analyses

We investigated for both the AUD and control group whether there were any changes in connectivity between timepoint 1 and 2. Furthermore, we investigated if there were any differences between the two groups related to the changes in connectivity between these two time points. Connectivity changes for the networks of interest (i.e., DMN, right FPN and left FPN) were studied at two levels: (1) whole brain connectivity and (2) within network connectivity.

We used the well described network templates that were identified by Smith and colleagues (2009) (10) to study the connectivity of our networks of interest (see Supplementary Figure S1 for the spatial maps of these networks). Smith and colleagues (10) identified these network templates by performing independent component analysis (ICA). ICA is a powerful data-driven approach that can decompose an fMRI dataset into temporally coherent, spatially independent components, which correspond to major functional brain networks (11).

#### 2.5.1. Whole brain functional connectivity

The whole brain connectivity of our networks of interest, reflecting the connectivity of these networks with themselves and the rest of the brain, was investigated using dual regression. We applied the unthresholded group ICA maps of all 20 components from Smith and colleagues 2009 as spatial maps into dual regression (10). Dual regression uses these spatial maps as input to generate subject-wise time courses for these networks by correlating the mean time course of each network with all the voxels of the brain. Regression of these time courses against the data resulted in spatial maps of the 20 networks for each individual participant (40). Afterwards, we selected the spatial maps for our three networks of interest (i.e., the DMN, left FPN, and right FPN).

The spatial maps resulting from dual regression were subtracted (timepoint 2 minus timepoint 1) to investigate the effects of time and the differences between the AUD and control group. We used permutation tests via randomize (10,000 permutations) for inference testing (41). The results from these tests were considered significant using a threshold-free cluster enhancement corrected value of *p* of 0.05 (42) and a minimum cluster size of 5 voxels.

#### 2.5.2. Within network functional connectivity

For the within network connectivity analyses we generated a mask for the networks of interest by thresholding (*Z* ≥ 3) the statistical maps of each network [selected from the Smith and colleagues’ templates (10)]. These masks were used to extract the mean within network connectivity in both resting-state scans (i.e., timepoint 1 and 2), from the individual spatial maps generated in the dual regression procedure. This approach results in one value per participant and network, which represents an aggregate measure of mean within network connectivity. The effect of time (abstinence in the patient group) was investigated with a paired comparison between the connectivity strength between timepoint 1 and timepoint 2, using a Wilcoxon signed-rank test (we used this non-parametric test as the data (for AUD patients and controls) was not normally distributed). The differences between the controls and AUD group were calculated using the Mann–Whitney U test (non-normal distribution) on the difference scores (timepoint 2 minus 1) (alpha = 0.05).

### 2.6. Measures of interest

Various measures were collected for exploratory correlational analyses (see “2.7 Statistical analysis” below). On the days of the MRI scans (i.e., timepoint 1 and 2), we collected measures for ‘working memory’ [maximum number of reproduced digits on the Letter-number sequencing task (43)], and cognitive flexibility [sum scores for perseverative responses and perseverative errors on the Wisconsin Card Sorting Test (44, 45)]. We included these measures for executive functioning, as there are clear indications for impairments in these functions in AUD, and that these impairments may be related to treatment compliance and everyday functioning (46, 47). Furthermore, during the first week of admission, we collected measures related to trait anxiety, impulsivity, and alcohol use. Trait anxiety was measured with the Spielberger State–Trait Anxiety Inventory-Y2 (STAI-Y2) (48, 49), and (choice) impulsivity with the delay discounting task (50). These measures were included, since studies have shown increased levels of trait anxiety (51, 52) and impulsivity (53, 54) in AUD, which are clinically relevant and may be predictive of relapse (53–55). Alcohol use was quantitated *via* the sum score on the Alcohol Use Disorder Identification Test (AUDIT) (56), and the number of standard drinks in the 30 days preceding admission [measured with the Alcohol Timeline Followback (57)]. Finally, we used the Lifetime Drinking History (LDH) to measure the total number of lifetime drinks, age of first drink, and heavy drinking years [periods of time in which individuals drank >6 standard drinks/day (in accordance with the LDH Manual)] (58). Importantly, the FPN and DMN have been implicated in abovementioned functions and measures related to executive functioning, trait anxiety, impulsivity, and the severity of alcohol use/AUD (3, 4, 12–15, 53, 59–62).

### 2.7. Statistical analysis

Exploratory correlational analyses were performed between any observed significant change in within network connectivity strength and the measures of interest described above. We performed Spearman correlations (non-normal distribution) in both the AUD and control groups separately (alpha = 0.05). Correlation coefficients were compared between AUD patients and controls by comparing the standardized correlation coefficients (Fisher’s r to z transform), using an ANOVA for summary data (alpha = 0.05). For the Wisconsin Card Sorting Test and Letter-number sequencing task we correlated the change in performance on these tasks with the change in network connectivity strength. For all these correlations, we performed supplemental partial correlations (controlling for years of education), in order to investigate if this affected the comparison between the AUD and control group [as these groups differed with respect to the years of education (see Results)].

In addition to the correlational analyses described above, we performed for both the Wisconsin Card Sorting Test and Letter-number sequencing task correlations between scores on these tasks at the day of scanning and the network connectivity strength at the same day separately, as a supplemental analysis. These analyses were performed separately, since the change score on these tasks may be more difficult to interpret, as a change in these scores may result from a combination of recovery related to the early abstinence period and the learning effect by performing the same task twice.

We tested for differences between the AUD and control group for the measures of interest described above (a repeated measures ANOVA for the Wisconsin card sorting test and Letter-number sequencing task, and a *t*-test or Mann–Whitney U test for the other measures). Finally, for the resting state scans Mann–Whitney U tests (non-normal distribution) were performed to test for potential differences in movement between the patient and control group (mean relative and absolute framewise displacement between successive images).

Finally, we would like to note that corrections for multiple comparisons were not primarily performed for either the fMRI analyses or the correlational analyses. We chose this exploratory set up as still little is known about the changes in functional connectivity in the early abstinence period in AUD, and how these potentially relate to neuropsychological and behavioral measures. When interpreting the results, it is important to keep in mind the more exploratory nature of these analyses. Finally, in case of significant fMRI or correlational results, we do also provide the Bonferroni corrected results in order to give more insight into the strength of these findings.

## 3. Results

### 3.1. Participants

In the present study we included 44 AUD patients, of which seven patients were excluded from the final analysis, because of too much movement (*n* = 6) or the resting-state scan not being available for both timepoints (*n* = 1). Of the 37 included AUD patients 22 were males. 27 healthy control participants (16 males) were included, that were matched with the patients with respect to age (*U* = 459.0, *p* = 0.581) and sex (*χ*^2^ = 0.00, *p* = 0.987). Patients and controls differed in years of education (*U* = 174.0, *p* < 0.001) (Table 1). All patients had current alcohol dependence according to the DSM-IV (*n* = 16) or alcohol use disorder according to DSM-5 (*n* = 21). Five (13.5%) of the AUD patients had one or more comorbid substance use disorder(s), with cannabis use disorder being the most prevalent comorbid substance use disorder [*n* = 4 (10.8%)]. Eighteen (48.6%) of the AUD patients had a comorbid psychiatric disorder other than a substance use disorder (with five patients (13.5%) having comorbid major depressive disorder and four (10.8%) an alcohol induced mood/depressive disorder; see Table 2 for an overview of all comorbid diagnoses).

**Table 1:** Demographics and clinical characteristics.

	Controls (*n* = 27)	AUD patients (*n* = 37)	Comparison between controls and AUD patients (*F*/*χ*^2^/*t*/*U*), Value of *p*
Demographics and general information			
Age (years), median (range)	47 (33–59)	47 (30–58)	*U* = 459.0, *p* = 0.581
Sex, %male (M/F)	59.3% (16/11)	59.5% (22/15)	*χ*^2^ = 0.00, *p* = 0.987
Years of education, median (range)	16 (12–26)	13 (4–18)	*U* = 174.0, *p* < 0.001**
Smokers (%)	0	51.4	*χ*^2^ = 19.72, *p* < 0.001**
Race
Asian	0	2	*χ*^2^ = 3.40, *p* = 0.493
Black/African American	9	14	
Multiracial	2	4	
White	15	17	
Unknown	1	0	
Time difference between scan 1 and 2 (days), median (range)	27 (21–35)	22 (19–31)	*U* = 277.0, *p* = 0.002**
Days between admission date and first scan, median (range)	N/A	6 (2–7)	-
Alcohol related measures			
Age of first drink, mean (SD)	16.19 (3.77)	14.63 (3.77)	*t* = 1.60, *p* = 0.114
Heavy drinking years, median (range)	0 (0–4)	13 (0–33)	*U* = 15.0, *p* < 0.001**
AUDIT sum score, median (range)	2 (0–6)	29 (15–38)	*U* = 0.0, *p* < 0.001**
Number of drinks in the 30 days preceding admission, median (range)	3 (0–24)	335 (45–1,100)	*U* = 0.0, *p* < 0.001**
Days since last drink (before first scan), median (range)	-	6 (2–12)	-
Other measures of interest			
Wisconsin card sorting test (mean, SD)			
Total number perseverative responses T1	15.07 (27.07)	20.54 (15.44)	Time effect: *F*(1,61) = 9.62, *p* = 0.003** Group effect: *F*(1,61) = 0.98, *p* = 0.327 Time by group interaction: *F*(1,61) = 0.10, *p* = 0.753
Total number perseverative responses T2	10.77 (17.71)	14.78 (16.14)	
Total number perseverative errors T1	12.70 (19.99)	18.68 (13.13)	Time effect: *F*(1,61) = 10.81, *p* = 0.002** Group effect: *F*(1,61) = 1.85, *p* = 0.179 Time by group interaction: *F*(1,61) = 0.35, *p* = 0.559
Total number perseverative errors T2	9.31 (13.25)	13.38 (13.48)	
Letter-number sequencing (mean, SD)			
Maximum number reproduced digits T1	7.93 (1.24)	7.30 (0.97)	Time effect: *F*(1,61) = 1.08, *p* = 0.302 Group effect: *F*(1,61) = 3.93, *p* = 0.052 Time by group interaction: *F*(1,61) = 0.50, *p* = 0.482
Maximum number reproduced digits T2	7.96 (1.26)	7.53 (1.03)	
Trait anxiety (STAI-Y2) (median, range)	26 (20–38)	49 (26–69)	*U* = 34.0, *p* < 0.001**
Delay discounting (ln k value) (median, range)^¥^	−5.13 (−6.32 – −0.13)	−4.02 (−8.52 – −0.45)	*U* = 363.5, *p* = 0.098

^¥^In delay discounting the factor *k* represents the rate of discounting of the delayed outcome. As *k* values are not normally distributed, a natural log-transformation is applied, and the *ln(k)* values are displayed in this table. Higher *ln(k)* values (i.e., less negative values) mean greater preference for immediate rewards. AUD, alcohol use disorder; AUDIT, Alcohol Use Disorder Identification Test; F, female; M, male; SD, standard deviation; STAI-Y2, Spielberger State–Trait Anxiety Inventory-Y2; *χ*^2^, Pearson’s chi square (2-tailed); *t*, independent *t*-test (2-tailed); *U*, Mann–Whitney-U test (2-tailed). *p < 0.05, **p < 0.01.

**Table 2:** Current psychiatric disorders according to the SCID-IV/SCID-5 interviews in the AUD patients.

Current diagnosis	Number of patients with this disorder in the AUD group (*n* = 37)
Current substance use disorders, *N* (%)	
Cannabis use disorder	4 (10.8%)
Cocaine/stimulant use disorder	2 (5.4%)
Opioid use disorder	0 (0%)
Sedative/hypnotic/anxiolytic use disorder	1 (2.7%)
Hallucinogen use disorder	0 (0%)
Other substance use disorder	0 (0%)
Mood/depressive disorders, *N* (%)	
Major depressive disorder	5 (13.5%)
Dysthymic/persistent depressive disorder	1 (2.7%)
Alcohol induced mood/depressive disorder	4 (10.8%)
Bipolar disorder	2 (5.4%)
Anxiety disorders, *N* (%)	
Generalized anxiety disorder	3 (8.1%)
Agoraphobia	0 (0%)
Social phobia/social anxiety disorder	2 (5.4%)
Specific phobia	0 (0%)
Anxiety disorder (not) otherwise specified	2 (5.4%)
Panic disorder	0 (0%)
Alcohol-induced anxiety disorder	0 (0%)
Trauma related disorders, *N* (%)	
Posttraumatic stress disorder	5 (13.5%)
Other specified trauma disorder	1 (2.7%)
Obsessive compulsive and related disorders, *N* (%)	
Obsessive compulsive disorder	0 (0%)
Neurodevelopmental disorder, *N* (%)	
Attention deficit hyperactivity disorder	4 (10.8%)

Reported as number of participants who met criteria for each disorder based on SCID-IV/SCID-5. Terminology related to substance use disorders reflects DSM-5 classification, in which DSM-IV substance abuse and dependence are combined into substance use disorders according to DSM-5. AUD, alcohol use disorder; SCID, Structured Clinical Interview for DSM.

As the AUD participants were participating in a treatment program, pharmacological interventions were started when appropriate. While none of the patients received pharmacotherapy for AUD at timepoint 1, at timepoint 2 eleven participants were treated with naltrexone and one with acamprosate. In addition, while only three patients received antidepressants at timepoint 1, thirteen patients received antidepressants at timepoint 2 (see Supplementary Table A for a more complete medication overview).

### 3.2. Behavioral results

The AUD patients showed higher trait anxiety levels than the controls (*U* = 34.0, *p* < 0.001) (Table 1; Supplementary Figure S2). In addition, the AUD patients showed a trend for a steeper delayed reward discounting compared to controls (*U* = 363.5, *p* = 0.098) (Table 1; Supplementary Figure S3), indicating a trend for a preference for smaller, immediate rewards over larger, delayed rewards.

### 3.3. Functional MRI

#### 3.3.1. Movement

There were no significant differences in movement between the AUD and control group with respect to the mean absolute (Timepoint 1: *U* = 411.0, *p* = 0.229, Timepoint 2: *U* = 438.0, *p* = 0.403) or relative framewise displacement (Timepoint 1: *U* = 393.0, *p* = 0.148, Timepoint 2: *U* = 392.0, *p* = 0.144) (Supplementary Table B).

#### 3.3.2. Default mode network

Neither the AUD patients (*Z* = −0.309, *p* = 0.757) nor the controls (*Z* = −0.336, *p* = 0.737) showed a significant change in within DMN connectivity between timepoint 1 and 2. There was also no difference between the patients and controls in the change in within DMN connectivity (*U* = 466, *p* = 0.649). Finally, the whole brain analysis did not show any significant results related to the change in DMN connectivity.

#### 3.3.3. Frontoparietal networks

**Figure 1:**
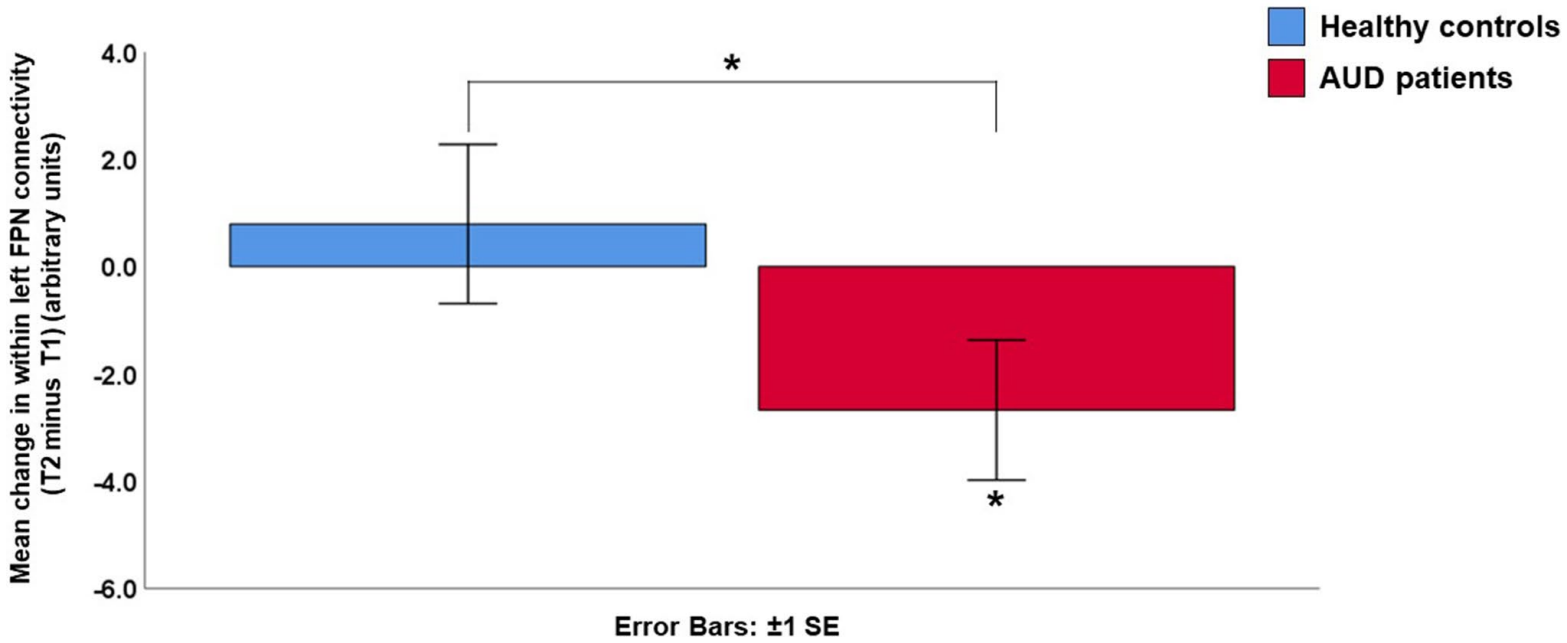
Mean change in within left frontoparietal network (FPN) connectivity strength (Timepoint 2 minus Timepoint 1). The alcohol use disorder (AUD) patients showed a significant decrease in within left frontoparietal network connectivity, which differed significantly from the control group. AUD, alcohol use disorder; FPN, frontoparietal network; SE, standard error; T1, timepoint 1; T2, timepoint 2.

While there were no significant results for the right FPN (AUD patients: *Z* = −0.249, *p* = 0.803, controls: *Z* = −0.745, *p* = 0.456, difference between AUD patients and controls: *U* = 457, *p* = 0.563), there were several significant results for the left FPN. The patients showed a decrease in left FPN connectivity strength at timepoint 2 compared to timepoint 1 (*Z* = −2.029, *p* = 0.042). Although the controls did not show a significant change in within left FPN connectivity (*Z* = −1.081, *p* = 0.280), the change in the left FPN connectivity in the patient group did differ significantly from the controls (*U* = 352, *p* = 0.045) (Figure 1; Supplementary Figure S4). *Post-hoc* tests showed that the within left FPN connectivity did not significantly differ between the patients and controls at time point 1 (*U* = 455, *p* = 0.545), or timepoint 2 (*U* = 380, *p* = 0.104) separately (Supplementary Figure S5). When Bonferroni correction was performed for multiple comparisons (i.e., value of *p*s multiplied by three, for the three networks that were investigated), the decrease in within left FPN connectivity in the AUD group (Bonferroni corrected value of *p* = 0.126) and the difference between the AUD and control group (Bonferroni corrected value of *p* = 0.135) were no longer significant.

**Figure 2:**
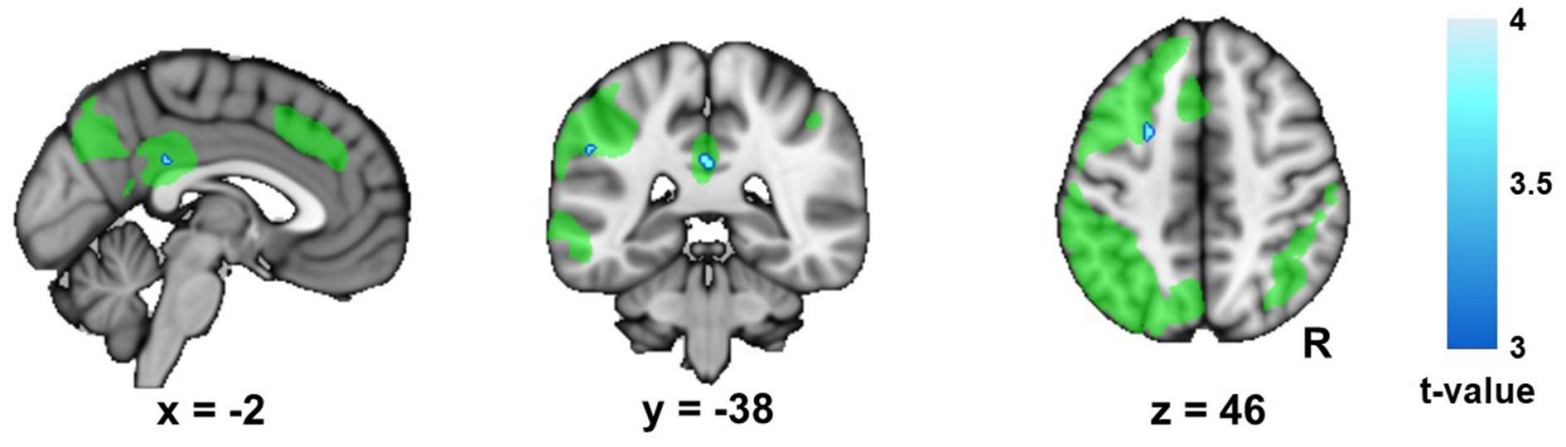
Change in left frontoparietal network (FPN) connectivity in the Alcohol Use Disorder (AUD) group. This figure displays the changes (timepoint 2 minus timepoint 1) in whole brain connectivity of the left frontoparietal network (FPN) in the alcohol use disorder (AUD) group. The left FPN template (Smith and colleagues 2009) is shown in green for display purposes, in order to show that the significant clusters are all located within this network. R, right.

The whole brain analyses revealed a decrease in left FPN connectivity in AUD patients at timepoint 2 compared to timepoint 1. The result confirmed the decrease in within left FPN connectivity, by revealing that all significant clusters were located within the left FPN template, with significant clusters located in the middle frontal gyrus, posterior parietal cortex and posterior cingulate cortex (Figure 2; Supplementary Table C). None of these clusters survived Bonferroni correction for the three networks that were being studied (Supplementary Table C). The controls did not show any significant changes in whole brain left FPN connectivity over time, nor were there differences between the patients and controls.

### 3.4. Correlational analyses

Table 3 shows the correlations between the change in within left FPN connectivity and measures of interest. Although there were no significant correlations, the AUD patients showed trends for associations of the change in left FPN connectivity with trait anxiety and delay discounting. The AUD patients showed a trend for a positive association between the decrease in left FPN connectivity and trait anxiety (r_s_ = 0.303, *p* = 0.068), indicating that lower trait anxiety was associated with a larger change (larger decrease from timepoint 1 to timepoint 2) in left FPN connectivity. In addition, the AUD patients showed a trend for a negative association between the decrease in left FPN connectivity and delay discounting (r_s_ = −0.283, *p* = 0.089), meaning that higher (i.e., less negative) discounting scores (indicating a greater preference for immediate rewards) are associated with a larger decrease in left FPN connectivity. These patterns did not differ between the patients and controls (trait anxiety: *F*(1,63) = 1.149, *p* = 0.288; delay discounting: *F*(1,62) = 0.507, *p* = 0.479) (Table 3). Our supplemental partial correlations showed that this pattern did also not differ between AUD patients and controls when years of education were taken into account (trait anxiety: *F*(1,62) = 1.033, *p* = 0.313; delay discounting: *F*(1,61) = 0.003, *p* = 0.956). The partial correlations were no longer at a trend level in the AUD group for trait anxiety (*r* = 0.194, *p* = 0.265) or delay discounting (*r* = −0.175, *p* = 0.314) (Supplementary Table D). Finally, there were no significant relationships between the left FPN connectivity and the performance on the Wisconsin card sorting test or Letter-number sequencing task, nor related to changes in performance (Table 3), nor with respect to correlations for each scan day separately (Supplementary Table E).

**Table 3:** Correlational analyses for the relation between the change in within left frontoparietal network connectivity (T2 minus T1) and the measures of interest.

	Change in within left FPN connectivity in controls (T2 minus T1)	Change in within left FPN connectivity in AUD patients (T2 minus T1)	Comparison between controls and AUD patients for standardized correlation coefficient (*F*, value of *p*)^‡^
Change in Wisconsin card sorting test score (T2 minus T1)						
Total number of perseverative responses	r_s_ = −0.282, *p* = 0.163 z_r_ = −0.290, SE_zr_ = 0.209	r_s_ = 0.189, *p* = 0.263 z_r_ = 0.191, SE_zr_ = 0.172	*F*(1,62) = 3.198, *p* = 0.079
Total number of perseverative errors	r_s_ = −0.271, *p* = 0.181 z_r_ = −0.278, SE_zr_ = 0.209	r_s_ = 0.224, *p* = 0.183 z_r_ = 0.228, SE_zr_ = 0.172	*F*(1,62) = 3.534, *p* = 0.065
Change in Letter-number sequencing score (maximum number of reproduced digits) (T2 minus T1)	r_s_ = −0.004, *p* = 0.986 z_r_ = −0.004, SE_zr_ = 0.204	r_s_ = 0.059, *p* = 0.733 z_r_ = 0.059, SE_zr_ = 0.174	*F*(1,62) = 0.056, *p* = 0.814
Trait anxiety (STAI-Y2)	r_s_ = 0.028, *p* = 0.891 z_r_ = 0.028, SE_zr_ = 0.204	r_s_ = 0.303, *p* = 0.068 z_r_ = 0.313, SE_zr_ = 0.172	*F*(1,63) = 1.149, *p* = 0.288
Delay discounting (ln(k) score)	r_s_ = −0.099, *p* = 0.629 z_r_ = −0.099, SE_zr_ = 0.209	r_s_ = −0.283, *p* = 0.089 z_r_ = −0.291, SE_zr_ = 0.172	*F*(1,62) = 0.507, *p* = 0.479
Age of first drink (years)	r_s_ = −0.221, *p* = 0.277 z_r_ = −0.225, SE_zr_ = 0.209	r_s_ = −0.060, *p* = 0.732 z_r_ = −0.060, SE_zr_ = 0.177	*F*(1,60) = 0.364, *p* = 0.548
Total number of lifetime drinks	r_s_ = 0.279, *p* = 0.187 z_r_ = 0.287, SE_zr_ = 0.218	r = −0.039, *p* = 0.825 z_r_ = −0.039, SE_zr_ = 0.177	*F*(1,58) = 1.355, *p* = 0.249
Heavy drinking years	N/A	r_s_ = −0.104, *p* = 0.554 z_r_ = −0.104, SE_zr_ = 0.177	N/A
AUDIT score	r_s_ = 0.041, *p* = 0.838 z_r_ = 0.041, SE_zr_ = 0.204	r_s_ = 0.076, *p* = 0.653 z_r_ = 0.076, SE_zr_ = 0.172	*F*(1,63) = 0.017, *p* = 0.895
Number of drinks past 30 days before admission	r_s_ = 0.070, *p* = 0.730 z_r_ = 0.070, SE_zr_ = 0.204	r_s_ = 0.055, *p* = 0.751 z_r_ = 0.055, SE_zr_ = 0.174	*F*(1,62) = 0.003, *p* = 0.956

AUDIT, Alcohol Use Disorder Identification Test; FPN, frontoparietal network; N/A, not applicable; r_s_, Spearman’s correlation coefficient; SE_zr_, standard error of the normalized correlation coefficient; STAI-Y2, Spielberger State–Trait Anxiety Inventory-Y2; T1, timepoint 1; T2, timepoint 2; z_r_, standardized correlation coefficient. ^‡^*F*-test comparing the standardized correlation coefficient between the AUD patients and controls.

### 3.5. *Post-hoc* analyses

*Post-hoc* analyses were performed to investigate if the change in left FPN connectivity (timepoint 2 minus timepoint 1) differed between the AUD patients that were treated with antidepressants or medication for AUD (i.e., naltrexone or acamprosate) and the patients that were not treated with such medication (using the Mann–Whitney U test). No patients received medication for AUD at timepoint 1 and twelve patients received such medication at timepoint 2. The change in left FPN connectivity did not differ between these twelve AUD patients that did receive medication for AUD and the 25 patients that did not receive such medication (*U* = 105.0, *p* = 0.151). All three patients that were treated with antidepressants at timepoint 1 were still receiving antidepressants at timepoint 2. In total thirteen patients were treated with antidepressants at timepoint 2. There was no difference in the change of within left FPN connectivity between these thirteen patients on antidepressants and the 24 patients that did not receive antidepressant medication (*U* = 131.0, *p* = 0.441).

## 4. Discussion

In this study we investigated longitudinal changes in resting-state connectivity during the early abstinence period in AUD on a large-scale network level. In contrast to the controls, who showed no longitudinal change, the AUD group showed a decrease in within left FPN connectivity during the follow-up period. The results from the whole brain connectivity analysis further confirmed these results, since all clusters that showed a decrease in left FPN connectivity during the early abstinence period in AUD were located within this network itself. However, these results for the left FPN did not survive a strict Bonferroni correction for multiple comparisons. Finally, our exploratory correlational analyses revealed a trend for an association of the change in within left FPN connectivity with trait anxiety and delay discounting in AUD.

The decrease in within left FPN connectivity in the early abstinence period in AUD is a novel finding, as little is known about this critical period. Interestingly, our original hypothesis was that the FPN connectivity would increase during the early abstinence period. As still little is known about changes in resting-state functional connectivity during the early abstinence period, this hypothesis was based on studies after longer durations of abstinence. Camchong and colleagues showed increased resting-state connectivity of the FPN in long-term abstinent AUD patients (average of 7.91 years abstinence) compared to patients who were abstinent for a shorter time period (72.59 days abstinence) (14). It has been suggested that this upregulated FPN after long-term abstinence is a compensatory mechanism to facilitate top-down control (14, 20). We hypothesized that our longitudinal data would show an increase in resting-state FPN connectivity in AUD during the early abstinence period, reflecting an initial step in the process of upregulating the FPN connectivity. However, our findings suggest that the early abstinence period may be characterized by different changes in resting-state connectivity than the changes taking place after long-term abstinence. This highlights the importance of studying different phases of recovery from AUD, as this may further our understanding of the dynamic changes taking place during different phases in the recovery process.

Our results emphasize the importance of performing longitudinal studies, as the change in left FPN connectivity was the most sensitive measure for finding subtle changes in connectivity (both within the AUD group and when comparing the change in connectivity between the AUD patients and controls). As the left FPN connectivity did not differ between AUD patients and controls on timepoint 1 or 2 separately, there is no clear increased or decreased connectivity in AUD compared to health at either of these time points. The decrease in left FPN connectivity in the AUD group over time indicates that the AUD patients have relatively stronger left FPN connectivity at timepoint 1 (shortly after stopping alcohol) compared to timepoint 2. Below we discuss possible explanations for the change in the left FPN connectivity based on what is known from the literature, as this network is implicated in higher-order cognitive processes, emotion regulation and top-down control (12, 15, 61, 62). When we formulate hypotheses below about the left FPN being upregulated in AUD, then this refers to the hypothesis that the FPN is upregulated in AUD at timepoint 1 relative to timepoint 2 (and not compared to the controls).

The decrease in within left FPN connectivity in AUD may signify that this network is initially upregulated and recovers to a lower resting-state connectivity level over the early abstinence period. This could reflect that the FPN is upregulated over a longer time period for top-down control (63) in order to compensate for the effects of alcohol, with the FPN connectivity decreasing again when patients are abstinent. Alternatively, this result may reflect a more dynamic (short-term) pattern, with the left FPN only being upregulated in AUD shortly after patients stop alcohol consumption. It is clinically well-known that stopping alcohol consumption has anxiogenic effects. The negative affective state that arises when alcohol use is stopped is a negative reinforcer that can trigger relapse (2). From this perspective, the relatively higher left FPN connectivity immediately after stopping alcohol consumption may be an adaptive response to facilitate emotion-regulation and inhibitory control (15) in order to cope with negative affect and tendencies to use alcohol again (2). Finally, the FPN may also be involved more intrinsically in higher-order cognitive processes that arise after stopping alcohol consumption, for example related to problem solving attempts (64), repetitive negative thoughts (65), or thoughts about alcohol, which may diminish in the course of the early abstinence period.

There were no significant correlations between the changes in left FPN connectivity and our neuropsychological and behavioral measures of interest. The AUD patients showed trends for a relationship of the change in left FPN connectivity with trait anxiety and delay discounting. Below we describe these trends and what they might mean in the context of what is known from the literature. However, given the trend level of these results, the interpretations of these results can best be seen as hypotheses regarding what the neural changes might mean on the psychological level. Future studies should investigate if longer treatment/abstinence periods and larger sample sizes may provide sufficient power to drive these trends to a statistically significant level.

The AUD patients showed higher trait anxiety levels than the controls and within the AUD group there was a trend for a positive association between change in left FPN connectivity and trait anxiety. So, patients with lower trait anxiety have a relatively stronger left FPN connectivity directly after stopping alcohol consumption, that decreases during this early abstinence period. Interestingly, numerous studies have shown that the ability to regulate emotions in high-risk situations for relapse in alcohol use are important for relapse prevention (66). It may be a crucial factor to allocate resources to the FPN for emotion regulation (15) when needed, like in the stressful phase just after stopping alcohol consumption (2). Thus, our results may suggest that resources are allocated to the FPN for top-down control over anxiety directly after stopping with drinking, with FPN connectivity recovering to a lower resting-state connectivity level during the early abstinence period.

Our results showing a trend for steeper delayed reward discounting in AUD are consistent with the observation that smaller, immediate rewards are valued over larger, delayed rewards in AUD patients compared to controls (54). Importantly, steeper discounting serves as a robust measure for (choice) impulsivity (23, 54, 67) and has been associated with relapse risk across various addiction disorders, including AUD (53, 54). In addition, impulsivity, with the inability to inhibit alcohol consumption despite negative consequences, is a core aspect of the addiction cycle (66). We observed a trend for a negative association between change in left FPN connectivity and delay discounting. Previous studies in AUD and various other addiction disorders have shown higher activity in the FPN during choices for larger, delayed rewards relative to choices for smaller, immediate rewards (53). This may suggest that patients with addiction disorders need to recruit greater neural resources to exert restraint and chose for larger, delayed rewards. In line with this, our results may suggest that in patients with a steeper discounting curve the FPN may be upregulated more strongly after stopping alcohol use, which may serve as a compensatory mechanism to resist impulses for more immediate gratification and alcohol use.

Although earlier studies have found differences in the DMN connectivity between AUD patients and healthy controls, still little is known about potential longitudinal changes in this network (4, 17, 21). Importantly, we did not find any changes in DMN connectivity during the early abstinence period. This may mean that the DMN connectivity is more stable over time in AUD. However, we cannot exclude that changes in DMN connectivity take place over longer abstinence periods. Furthermore, we investigated the DMN as a whole and opposite changes at the DMN subsystem level may cancel each other out on the large-scale systems level. Seed based analyses may be more sensitive for finding potential changes in the specific DMN subsystems, since previous studies have shown that resting-state functional connectivity of the anterior DMN (which is involved in emotion regulation) tends to be decreased in addiction, whereas the resting state connectivity of the posterior DMN (which directs attention to the internal world) tends to be increased (4). Future studies should further investigate these hypotheses.

The main strength of our study is that it is the first to longitudinally investigate changes in functional network connectivity during the early abstinence period on a large-scale network level. However, our study has to be interpreted in the light of some limitations. First, our results did not survive a strict Bonferroni correction for multiple corrections. This may be related to the relatively small sample in our exploratory study, making it necessary to replicate our findings in larger samples. Second, although we do formulate hypotheses about what our neuroimaging results may mean on the psychological level, our trends for correlations do not allow us to make causal inferences about this with certainty. Future interventional studies (e.g., neurostimulation studies) should further investigate these hypotheses. Third, in the AUD group there were various comorbid disorders, related to substance use disorders, and other psychiatric diagnoses like major depressive disorder. While the presence of comorbidity may have influenced the observed results, comorbidity is common in AUD in clinical practice (68). Although a sample with AUD, without any comorbidity, may give more specific results, such a sample may raise concerns about the generalizability of results to general clinical populations. Studies in AUD samples both with and without comorbidity are needed, as they can complement each other. In addition, future studies in larger samples could provide opportunities for subgroup analyses, to study the effects of specific patterns of comorbidity. Fourth, while we did not find a difference in the change of within left FPN connectivity between the AUD patients that received medication (i.e., antidepressant medication, or medication for AUD) and the ones who did not, the (sub)groups in these analyses were small (with, e.g., only thirteen patients receiving antidepressants). In addition, the current study was not designed to investigate the effects of specific types of medication on brain function. The patients were studied in a standard treatment setting, in which pharmacological treatments were available according to individual needs. Pharmacological treatments were neither randomized across the AUD patients nor prescribed according to a standardized scientific protocol. Future studies should investigate potential pharmacological effects on functional connectivity in the early abstinence period in AUD in a more controlled study design, for example by comparing the effects of a pharmacological intervention with a placebo. Finally, while our correlational analyses provide tentative hypotheses about the meaning of our results on the psychological level, we did not collect state related measures with respect to anxiety, impulsivity, or (obsessive) thoughts about alcohol on the day of scanning itself. Future studies should include such measures to further test the hypotheses discussed above and investigate if state related measures may be more sensitive for finding associations.

Taken together, our results provide initial insight into the changes in large-scale network connectivity during the early abstinence period in AUD. Our results revealed a decrease in within left FPN connectivity during this phase in AUD patients. This result may reflect that the FPN is initially upregulated after stopping alcohol consumption and recovers to a lower resting-state connectivity level during the subsequent weeks of abstinence. We hypothesize that an initially upregulated FPN is an adaptive response to facilitate top-down control over anxiety and negative emotions, that are common after stopping alcohol use. This initially upregulated FPN may also help to control impulsive tendencies, which are a major risk factor for relapse. However, it is important to note that none of the neuroimaging results survived strict Bonferroni correction for multiple comparisons and the trends for correlations that were found were at uncorrected levels. Still, these initial results related to the clinically crucial early abstinence period could inform future study designs and encourage further studies on the dynamic neuroadaptations during this key period.

## Data availability statement

The raw data supporting the conclusions of this article will be made available in accordance to the Data Management & Sharing Policy set forth by the National Institute on Alcohol Abuse and Alcoholism and The National Institutes of Health.

## Ethics statement

This study involves human participants and was reviewed and approved by the NIH Institutional Review Board. All participants signed informed consent before participation.

## Author contributions

JO and RM contributed to the conception and design of the study and/or analyses approach. MS and RM organized the database. JO performed the statistical analyses and wrote the first draft. ND, DGe, YH, MS, DGo, and RM wrote sections of the manuscript. All authors contributed to manuscript revision, read, and approved the submitted version.

## Funding

This research was funded by NIAAA Intramural program (ZIAAA000125, PI: RM; and ZAIAA000213, PI: ND).

## Conflict of interest

The authors declare that the research was conducted in the absence of any commercial or financial relationships that could be construed as a potential conflict of interest.

## Publisher’s note

All claims expressed in this article are solely those of the authors and do not necessarily represent those of their affiliated organizations, or those of the publisher, the editors and the reviewers. Any product that may be evaluated in this article, or claim that may be made by its manufacturer, is not guaranteed or endorsed by the publisher.

## Supplementary material

The Supplementary material for this article can be found online at: https://www.frontiersin.org/articles/10.3389/fpsyt.2023.1185770/full#supplementary-material

SUPPLEMENTARY FIGURE S1 Networks of interest. We studied the connectivity of our networks of interest (i.e. the default mode network (DMN), left frontoparietal network (FPN), and right FPN) using dual regression. For this purpose, we used the well described network templates that were identified by Smith and colleagues (2009) using independent component analysis (ICA). Here, we display the spatial maps of these networks that were identified by Smith and colleagues (2009) (thresholded (*z* ≥ 3) for display purposes). Abbreviation: R: right.

SUPPLEMENTARY FIGURE S2 Trait anxiety in the healthy controls and alcohol use disorder patients. This dot plot displays the trait anxiety scores (sum score on the STAI-Y2) for the healthy controls and the alcohol use disorder (AUD) patients. The AUD patients showed higher trait anxiety levels than the controls (*U* = 34.0, *P* < 0.001). Abbreviations: AUD: alcohol use disorder, STAI-Y2: Spielberger State-Trait Anxiety Inventory-Y2.

SUPPLEMENTARY FIGURE S3 Delay discounting in the healthy controls and alcohol use disorder patients. This dot plot displays the delay discounting scores for the healthy controls and the alcohol use disorder (AUD) patients. The AUD patients showed a trend for a steeper delayed reward discounting compared to the controls (*U* = 363.5, *P* = 0.098). In delay discounting the factor *k* represents the rate of discounting of the delayed outcome. As *k* values are not normally distributed, a natural log-transformation is applied, and the *ln(k)* values are displayed in this figure. Higher *ln(k)* values (i.e. less negative values) mean greater preference for immediate rewards. Abbreviations: AUD: alcohol use disorder.

SUPPLEMENTARY FIGURE S4 Change in within left frontoparietal network connectivity. This dot plot displays the change in within left frontoparietal network (FPN) connectivity in the healthy controls and alcohol use disorder (AUD) patients (Timepoint 2 minus Timepoint 1). The alcohol use disorder (AUD) patients showed a significant decrease in within left FPN connectivity, which differed significantly from the control group (see also main text and Figure 1). Abbreviations: AUD: alcohol use disorder, FPN: frontoparietal network, T1: timepoint 1, T2: timepoint 2.

SUPPLEMENTARY FIGURE S5 Within left frontoparietal network connectivity at timepoint 1 and timepoint 2. These dot plots display the within left frontoparietal network (FPN) connectivity in the healthy controls and alcohol use disorder (AUD) patients at **(A)** Timepoint 1, and **(B)** Timepoint 2. The within left FPN connectivity did not significantly differ between the patients and controls at time point 1, or timepoint 2. Abbreviations: AUD: alcohol use disorder, FPN: frontoparietal network, T1: timepoint 1, T2: timepoint 2.
