# Supplementary figures and images for: Preliminary evidence for changes in frontoparietal network connectivity in the early abstinence period in alcohol use disorder: a longitudinal resting-state functional magnetic resonance imaging study
Source: Front Psychiatry. 2023 Jul 28;14:1185770. doi: 10.3389/fpsyt.2023.1185770 (PMC10420071; doi:10.3389/fpsyt.2023.1185770)

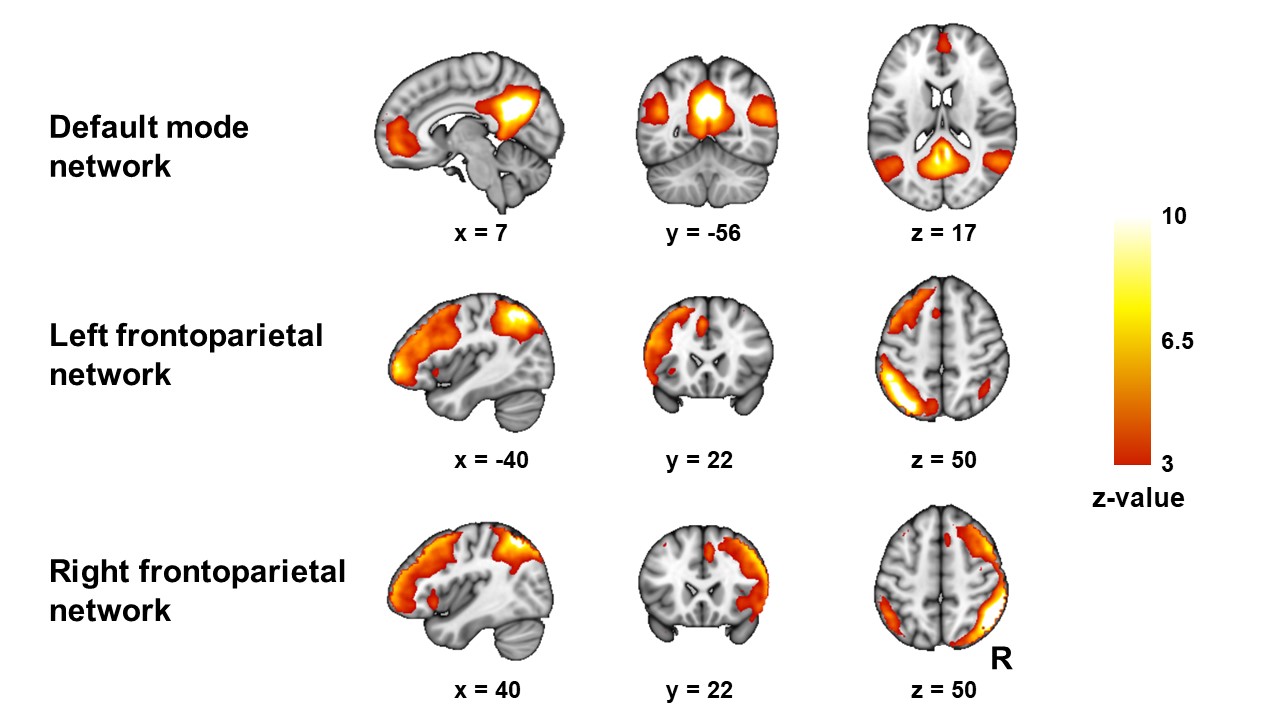

Supplement: SUPPLEMENTARY FIGURE S1 — Networks of interest. We studied the connectivity of our networks of interest (i.e. the default mode network (DMN), left frontoparietal network (FPN), and right FPN) using dual regression. For this purpose, we used the well described network templates that were identified by Smith and colleagues (2009) using independent component analysis (ICA). Here, we display the spatial maps of these networks that were identified by Smith and colleagues (2009) (thresholded (z ≥ 3) for display purposes). Abbreviation: R: right. [file Image_1.jpeg]

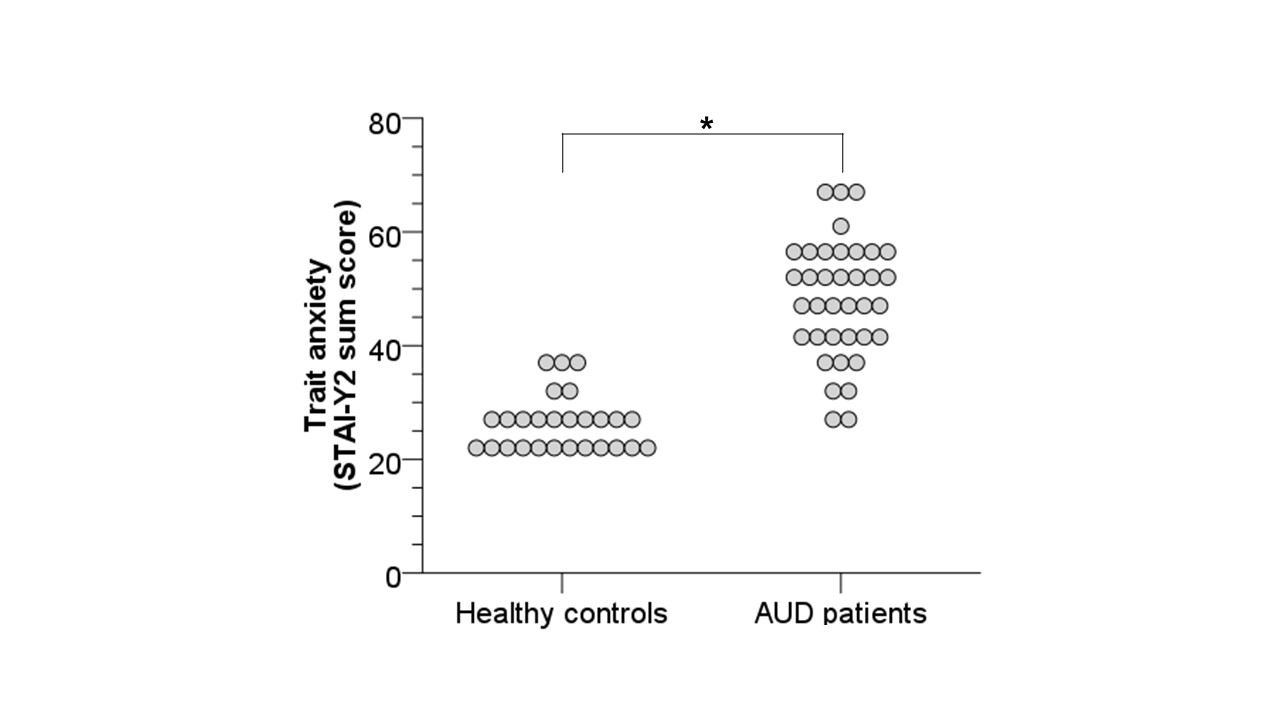

Supplement: SUPPLEMENTARY FIGURE S2 — Trait anxiety in the healthy controls and alcohol use disorder patients. This dot plot displays the trait anxiety scores (sum score on the STAI-Y2) for the healthy controls and the alcohol use disorder (AUD) patients. The AUD patients showed higher trait anxiety levels than the controls (U = 34.0, P < 0.001). Abbreviations: AUD: alcohol use disorder, STAI-Y2: Spielberger State-Trait Anxiety Inventory-Y2. [file Image_2.jpeg]

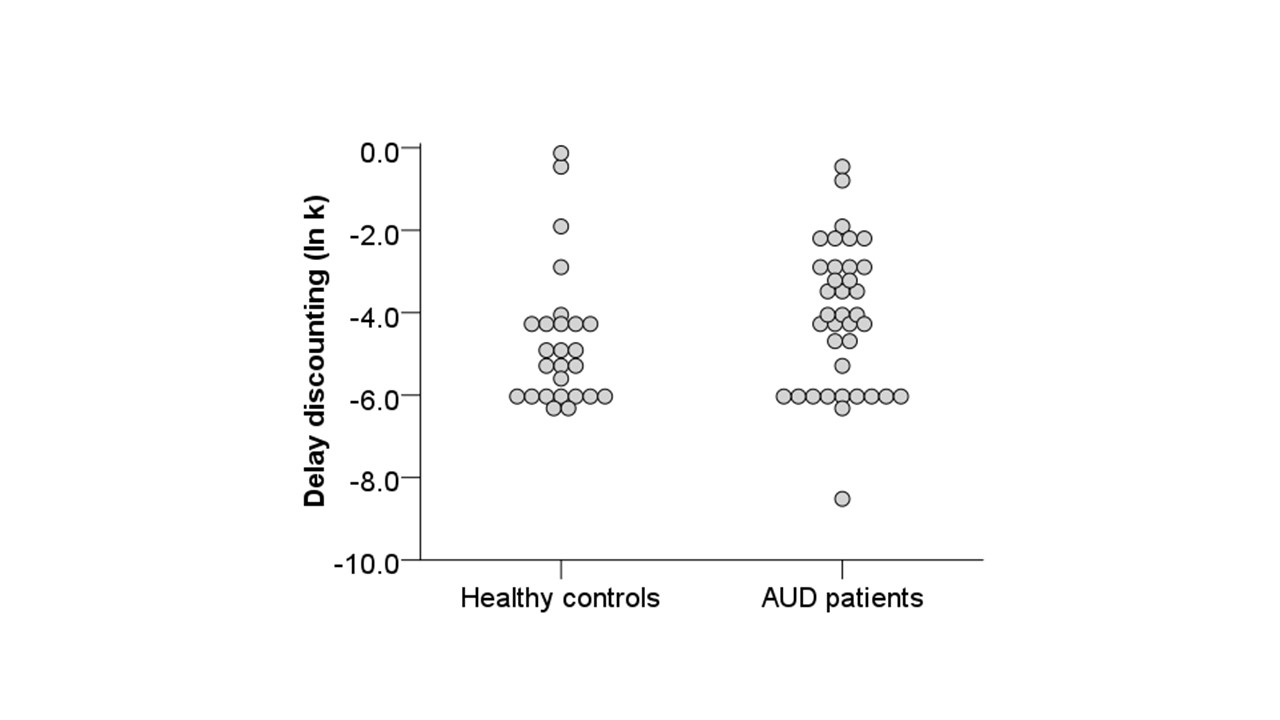

Supplement: SUPPLEMENTARY FIGURE S3 — Delay discounting in the healthy controls and alcohol use disorder patients. This dot plot displays the delay discounting scores for the healthy controls and the alcohol use disorder (AUD) patients. The AUD patients showed a trend for a steeper delayed reward discounting compared to the controls (U = 363.5, P = 0.098). In delay discounting the factor k represents the rate of discounting of the delayed outcome. As k values are not normally distributed, a natural log-transformation is applied, and the ln(k) values are displayed in this figure. Higher ln(k) values (i.e. less negative values) mean greater preference for immediate rewards. Abbreviations: AUD: alcohol use disorder. [file Image_3.jpeg]

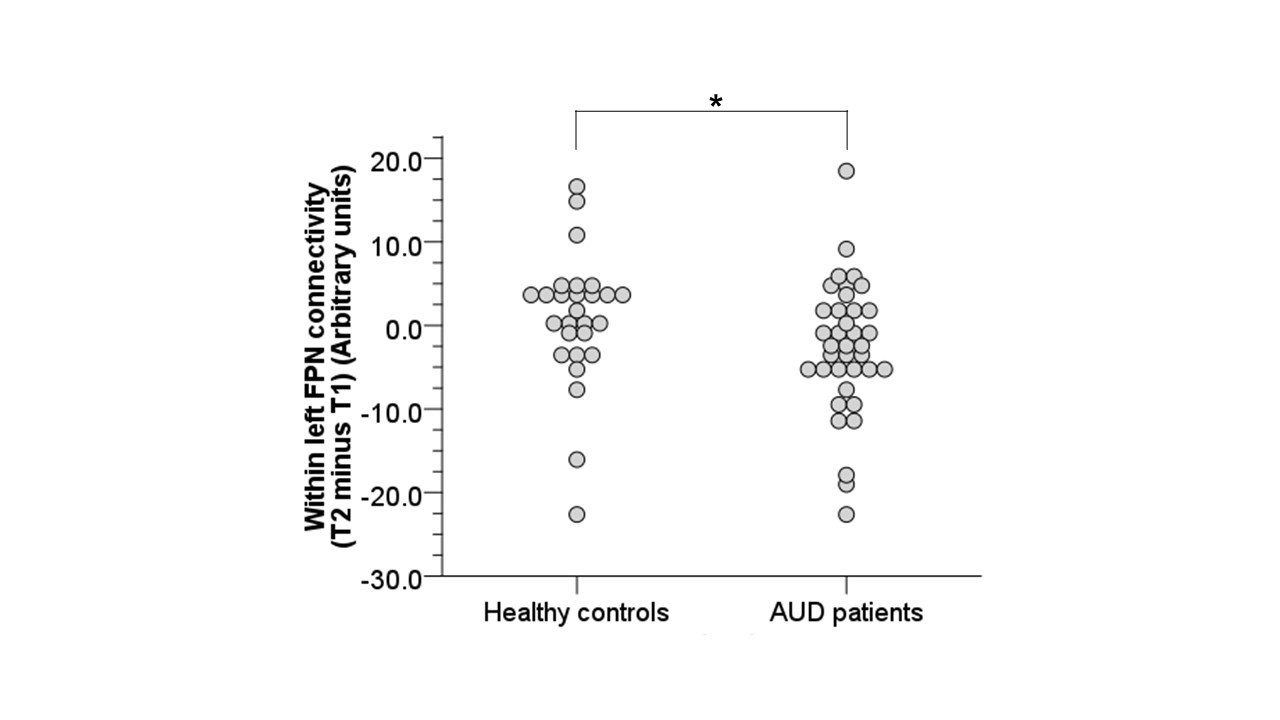

Supplement: SUPPLEMENTARY FIGURE S4 — Change in within left frontoparietal network connectivity. This dot plot displays the change in within left frontoparietal network (FPN) connectivity in the healthy controls and alcohol use disorder (AUD) patients (Timepoint 2 minus Timepoint 1). The alcohol use disorder (AUD) patients showed a significant decrease in within left FPN connectivity, which differed significantly from the control group (see also main text and Figure 1). Abbreviations: AUD: alcohol use disorder, FPN: frontoparietal network, T1: timepoint 1, T2: timepoint 2. [file Image_4.jpeg]

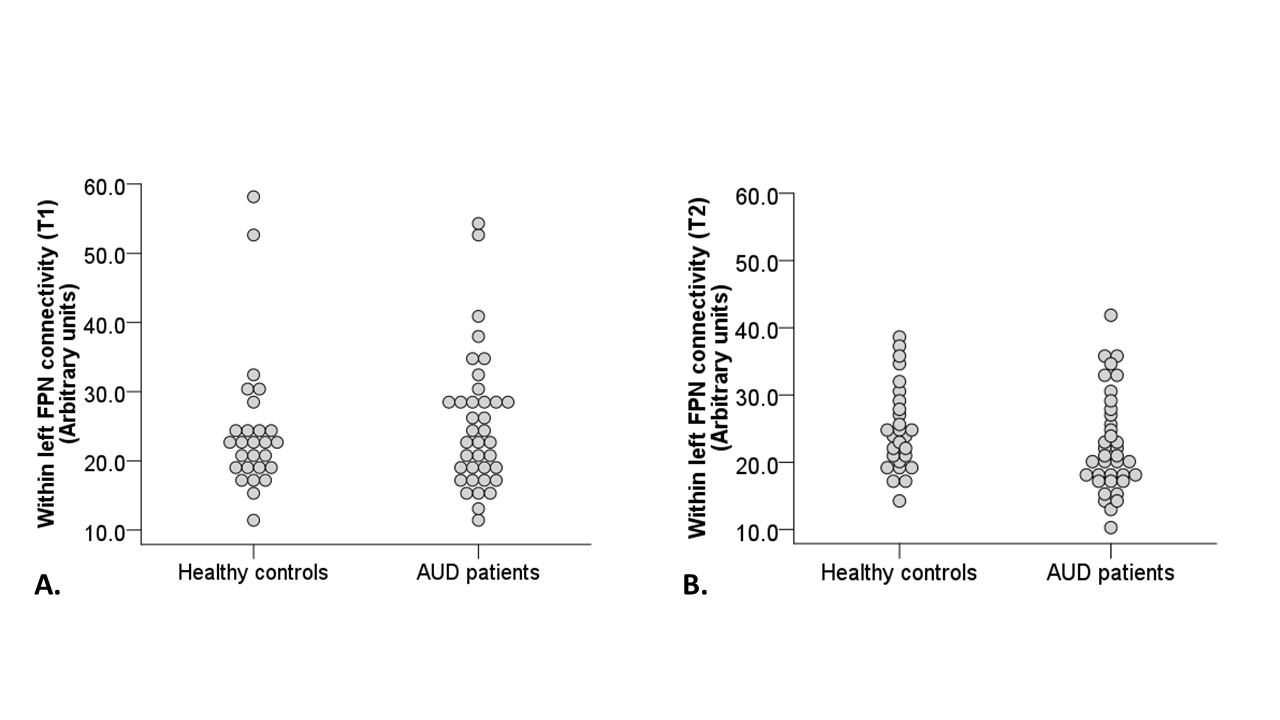

Supplement: SUPPLEMENTARY FIGURE S5 — Within left frontoparietal network connectivity at timepoint 1 and timepoint 2. These dot plots display the within left frontoparietal network (FPN) connectivity in the healthy controls and alcohol use disorder (AUD) patients at (A) Timepoint 1, and (B) Timepoint 2. The within left FPN connectivity did not significantly differ between the patients and controls at time point 1, or timepoint 2. Abbreviations: AUD: alcohol use disorder, FPN: frontoparietal network, T1: timepoint 1, T2: timepoint 2. [file Image_5.jpeg]
