# Supplementary material for: Preliminary evidence for changes in frontoparietal network connectivity in the early abstinence period in alcohol use disorder: a longitudinal resting-state functional magnetic resonance imaging study
Source: Front Psychiatry. 2023 Jul 28;14:1185770. doi: 10.3389/fpsyt.2023.1185770 (PMC10420071; doi:10.3389/fpsyt.2023.1185770)
Supplement: Supplementary file 6 [file Data_Sheet_1.PDF]

Supplemental Table A. Number of AUD patients using psychotropic drugs at the day of scanning

	AUD patients (n = 37)	
	**Timepoint 1**	**Timepoint 2**
**Medication use at day of scan (n = …)**[†]		
Naltrexone	0	11
Acamprosate	0	1
Antidepressant	3	13
Mood stabilizer	0	1
Antipsychotic	0	1
Stimulant	0	1
Benzodiazepines	2	0

[†] Number of patients using the described psychotropic medication at the day of scanning

Supplemental Table B. Movement during the resting-state scans

	Controls (n = 27)	AUD patients (n = 37)	Comparison between controls and AUD patients (U), p-value
**Mean absolute framewise displacement (in mm) (median, range)**			
Resting-state scan at visit 1	0.299 (0.100 – 1.415)	0.321 (0.096 – 2.389)	U = 411.0, p = 0.229
Resting-state scan at visit 2	0.391 (0.093 – 1.288)	0.358 (0.105 – 1.398)	U = 438.0, p = 0.403
**Mean relative framewise displacement (in mm) (median, range)**			
Resting-state scan at visit 1	0.083 (0.053 – 0.255)	0.124 (0.035 – 0.288)	U = 393.0, p = 0.148
Resting-state scan at visit 2	0.086 (0.042 – 0.283)	0.114 (0.028 – 0.274)	U = 392.0, p = 0.144

Abbreviations: AUD: alcohol use disorder, U: Mann-Whitney-U test (2-tailed)

Supplemental Table C. Changes in the left frontoparietal network connectivity in the early abstinence period (timepoint 2 minus timepoint 1) in the whole brain analysis in the Alcohol Use Diorder (AUD) group

Brain area	Hemisphere	Peak voxels (MNI coordinates)			Cluster size (# of voxels)	Significance level ($p_{uncorrected}$ = …)	Bonferroni corrected significance level ($p_{corrected}$ = …)‡
		X	Y	Z			
Cingular gyrus, posterior division	Ri	2	-38	26	12	0.037*	0.111
Supramarginal gyrus, parietal operculum cortex	Le	-52	-38	32	9	0.040*	0.120
Middle frontal gyrus	Le	-26	6	44	7	0.032*	0.096

Abbreviations: Le: left, Ri: right, MNI: Montreal Neurological Institute
‡: Bonferroni corrected results (for the three networks that are investigated).
* $p < 0.05$

Supplemental Table D. Partial correlation analyses (covariate: years of education) for the relation between changes in within left frontoparietal network connectivity (T2 minus T1) and measures of interest

	**Change in within Left FPN connectivity in controls (T2 minus T1)**	**Change in within Left FPN connectivity in AUD patients (T2 minus T1)**	**Comparison between controls and AUD patients for standardized correlation coefficient (F, p-value)‡**
**Change in Wisconsin card sorting test score (T2 minus T1)**			
Total number of perseverative responses	$r = 0.058$, $p = 0.783$<br>$z_r = 0.058$, $SE_{zr} = 0.224$	$r = 0.098$, $p = 0.575$<br>$z_r = 0.098$, $SE_{zr} = 0.183$	$F(1,61) = 0.019$, $p = 0.890$
Total number of perseverative errors	$r = 0.052$, $p = 0.805$<br>$z_r = 0.052$, $SE_{zr} = 0.224$	$r = 0.107$, $p = 0.542$<br>$z_r = 0.107$, $SE_{zr} = 0.183$	$F(1,61) = 0.037$, $p = 0.849$
**Change in Letter-number sequencing score (maximum number of reproduced digits) (T2 minus T1)**	$r = 0.073$, $p = 0.721$<br>$z_r = 0.073$, $SE_{zr} = 0.218$	$r = 0.034$, $p = 0.851$<br>$z_r = 0.034$, $SE_{zr} = 0.186$	$F(1,61) = 0.019$, $p = 0.892$
**Trait anxiety (STAI-Y2)**	$r = -0.091$, $p = 0.660$<br>$z_r = -0.091$, $SE_{zr} = 0.218$	$r = 0.194$, $p = 0.265$<br>$z_r = 0.197$, $SE_{zr} = 0.183$	$F(1,62) = 1.033$, $p = 0.313$
**Delay discounting (ln(k) score)**	$r = -0.160$, $p = 0.444$<br>$z_r = -0.161$, $SE_{zr} = 0.224$	$r = -0.175$, $p = 0.314$<br>$z_r = -0.177$, $SE_{zr} = 0.183$	$F(1,61) = 0.003$, $p = 0.956$
**Age of first drink (years)**	$r = 0.022$, $p = 0.916$<br>$z_r = 0.022$, $SE_{zr} = 0.224$	$r = 0.038$, $p = 0.833$<br>$z_r = 0.038$, $SE_{zr} = 0.189$	$F(1,59) = 0.003$, $p = 0.956$
**Total number of lifetime drinks**	$r = 0.029$, $p = 0.897$<br>$z_r = 0.029$, $SE_{zr} = 0.234$	$r = 0.300$, $p = 0.090$<br>$z_r = 0.310$, $SE_{zr} = 0.189$	$F(1,57) = 0.876$, $p = 0.353$
**Heavy drinking years**	N/A	$r = 0.006$, $p = 0.975$	N/A
**AUDIT score**	$r = -0.011$, $p = 0.959$<br>$z_r = -0.011$, $SE_{zr} = 0.218$	$r = 0.054$, $p = 0.759$<br>$z_r = 0.054$, $SE_{zr} = 0.183$	$F(1,62) = 0.053$, $p = 0.819$
**Number of drinks past 30 days before admission**	$r = -0.101$, $p = 0.623$<br>$z_r = -0.101$, $SE_{zr} = 0.218$	$r = 0.239$, $p = 0.174$<br>$z_r = 0.244$, $SE_{zr} = 0.186$	$F(1,61) = 1.461$, $p = 0.232$

AUDIT: Alcohol Use Disorder Identification Test, FPN: frontoparietal network, N/A: not applicable, r: partial correlation coefficient, $SE_{zr:}$: standard error of the normalized correlation coefficient, STAI-Y2: Spielberger State-Trait Anxiety Inventory-Y2, T1: timepoint 1, T2: timepoint 2, z_r: standardized correlation coefficient

‡: F-test comparing the standardized correlation coefficient between the AUD patients and controls

Supplemental Table E. Correlational analyses for the relation between the within left frontoparietal network connectivity (for T1 and T2 separately) and the measures of interest (for T1 and T2 separately)

		within Left FPN connectivity in controls (r_s , p-value)		within Left FPN connectivity in AUD patients (r_s , p-value)		Comparison between controls and AUD patients for standardized correlation coefficient (F, p-value)‡
		**T1**	**T2**	**T1**	**T2**	
**Wisconsin card sorting test**						
Total number of perseverative responses	**T1**	r_s = 0.134, p = 0.507<br>z_r = 0.135, SE_{zr} = 0.204		r_s = 0.042, p = 0.805<br>z_r = 0.042, SE_{zr} = 0.172		F(1,63) = 0.122, p = 0.728
	**T2**		r_s = -0.285, p = 0.158<br>z_r = -0.293, SE_{zr} = 0.209		r_s = -0.093, p = 0.585<br>z_r = -0.093, SE_{zr} = 0.172	F(1,62) = 0.551, p = 0.461
Total number of perseverative errors	**T1**	r_s = 0.157, p = 0.434<br>z_r = 0.158, SE_{zr} = 0.204		r_s = 0.012, p = 0.943<br>z_r = 0.012, SE_{zr} = 0.172		F(1,63) = 0.303, p = 0.584
	**T2**		r_s = -0.285, p = 0.158<br>z_r = -0.293, SE_{zr} = 0.209		r_s = -0.101, p = 0.554<br>z_r = -0.101, SE_{zr} = 0.172	F(1,63) = 0.508, p = 0.479
**Letter-number sequencing (maximum number of reproduced digits)**	**T1**	r_s = -0.157, p = 0.433<br>z_r = -0.158, SE_{zr} = 0.204		r_s = 0.037, p = 0.828<br>z_r = 0.037, SE_{zr} = 0.172		F(1,63) = 0.540, p = 0.465
	**T2**		r_s = -0.381, p = 0.050<br>z_r = -0.401, SE_{zr} = 0.204		r_s = 0.011, p = 0.949<br>z_r = 0.011, SE_{zr} = 0.174	F(1,63) = 2.371, p = 0.129

AUD: alcohol use disorder, SE_{zr}:: standard error of the normalized correlation coefficient, T1: timepoint 1, T2: timepoint 2, r_s: Spearman's correlation coefficient, z_r: standardized correlation coefficient

‡: F-test comparing the standardized correlation coefficient between the AUD patients and controls
